# In hospital risk factors for acute kidney injury and its burden in patients with Sars-Cov-2 infection: a longitudinal multinational study

**DOI:** 10.1038/s41598-022-07490-z

**Published:** 2022-03-02

**Authors:** Mario Luca Morieri, Claudio Ronco, Angelo Avogaro, Filippo Farnia, Marina Shestakova, Natalya Zaytseva, Natalya Mokrysheva, Larisa Akulkina, Anastasia Shepalina, Michail Brovko, Sergey Moiseev, Lucia Russo, Sara Mazzocut, Andrea Vianello, Anna Maria Cattellan, Monica Vedovato, Gian Paolo Fadini, Roberto Vettor, Paola Fioretto

**Affiliations:** 1 Department of Medicine (DIMED), University of Padova, Via Giustiniani 2, 35128 Padova, Italy; 2 International Renal Research Institute of Vicenza (IRRIV), Vicenza, Italy; 3 Endocrinology Research Center, Moscow, Russia; 4 Tareev Clinic of Internal Diseases, Sechenov First Moscow State Medical University, Moscow, Russia

**Keywords:** Kidney, Outcomes research

## Abstract

Acute kidney injury (AKI) is associated with increased mortality in most critical settings. However, it is unclear whether its mild form (i.e. AKI stage 1) is associated with increased mortality also in non-critical settings. Here we conducted an international study in patients hospitalized with SARS-CoV-2 infection aiming 1. to estimate the incidence of AKI at each stage and its impact on mortality 2. to identify AKI risk factors at admission (susceptibility) and during hospitalization (exposures) and factors contributing to AKI-associated mortality. We included 939 patients from medical departments in Moscow (Russia) and Padua (Italy). In-hospital AKI onset was identified in 140 (14.9%) patients, mainly with stage 1 (65%). Mortality was remarkably higher in patients with AKI compared to those without AKI (55 [39.3%] vs. 34 [4.3%], respectively). Such association remained significant after adjustment for other clinical conditions at admission (relative risk [RR] 5.6; CI 3.5- 8.8) or restricting to AKI stage 1 (RR 3.2; CI 1.8–5.5) or to subjects with AKI onset preceding deterioration of clinical conditions. After hospital admission, worsening of hypoxic damage, inflammation, hyperglycemia, and coagulopathy were identified as hospital-acquired risk factors predicting AKI onset. Following AKI onset, the AKI-associated worsening of respiratory function was identified as the main contributor to AKI-induced increase in mortality risk. In conclusion, AKI is a common complication of Sars-CoV2 infection in non-intensive care settings where it markedly increases mortality risk also at stage 1. The identification of hospital-acquired risk factors and exposures might help prevention of AKI onset and of its complications.

## Introduction

More than a year after SARS-CoV-2 was identified, the world is still facing significant health challenges. Infected patients mostly present mild symptoms and rapid recovery. Nevertheless, besides older age, male gender, diabetes (DM), and cardiovascular disease (CVD), decreased eGFR represents the strongest independent predictor of poor outcomes (longer hospital stay, long term effects, and mortality)^1,2^. Furthermore, acute kidney injury (AKI) has been reported to worsen the prognosis, being the most frequent condition reported in patients deceased with Sars-CoV-2 infection beyond respiratory failure^3,4^. The prevalence of AKI in COVID-19 studies is highly variable due to the heterogeneity of settings and the severity of the studied populations. AKI incidence exceeds 50% in intensive care unit (ICU) patients^5^, while in patients referred to non-intensive medical departments the rates are much lower (4 to 32%)^1,3,4^. A recent consensus document provides recommendations for the diagnosis, prevention, and management of COVID-19-associated AKI^6–8^. The majority of epidemiological studies report AKI rates and their association with mortality in critically-ill COVID-19 ICU patients focusing on severe AKI stages and their impact on outcomes. Information on less severe patients with mild AKI cases (stage I) admitted to non-ICU settings is lacking. Longitudinal studies evaluating exposures associated with the onset of AKI during hospitalization in COVID-19 patients as well as the impact of AKI at any stage on COVID-19 related mortality are also scant.

This multicenter international study aims at describing the incidence of AKI (any stage) in patients admitted to non-ICU medical departments (Padua, Italy, and Moscow, Russia) with COVID-19-associated pneumonia, and the relevant impact on mortality. We describe clinical and biochemical risk factors at admission (susceptibility) and hospital-acquired risk factors for AKI (exposures), and identify the conditions contributing to AKI-associated mortality in COVID-19 patients.

## Results

### Characteristics of the COVID-19 population and factors associated with AKI occurrence

The study included 939 patients hospitalized with Sars-CoV-2 infection. AKI was recorded in 140 (14.9%) subjects and occurred mainly within the first four days of hospitalization (median on day four, IQR 1–8); 91 (65%), 22 (16%), and 27 (19%) patients had AKI stage 1, 2, and 3, respectively. As shown in Table 1, AKI occurred more frequently in male and older patients. After accounting for these differences, AKI was more frequent among patients with diabetes, hypertension, and CKD (all p < 0.05), with no significant association with pre-hospitalization medications. Among symptoms and parameters at admission, AKI was associated with dyspnea, increased heart and respiratory rates, lower oxygen saturation, higher creatinine, fasting glucose levels, and C-reactive protein. When all these factors were analyzed together, age, male sex, diabetes, CKD, oxygen saturation, and CRP, were independently associated with AKI occurrence (supplementary table S2).

**Table 1:** Characteristics of COVID-19 patients according to AKI occurrence throughout the entire hospitalization. Data presented as mean (S.D.) or as percentage. In the column reporting data of all patients, data availability is also shown. MVA1: Model adjusted by age, sex, and clinical center.

	All patients N = 939		No AKI N = 799 (85%)	AKI N = 140 (15%)	P (MVA1)
	Available	Value	Value	Value	
Age, years	100%	62.0 ± 15.5	60.3 ± 15.3	71.6 ± 13.2	< 0.001
Sex male, %	100%	497 (52.9%)	402 (50.3%)	95 (67.9%)	< 0.001
**Concomitant risk factors, n (%)**					
Diabetes (Known + New)	100%	292 (31.1%)	235 (29.4%)	57 (40.7%)	0.014
Known Diabetes	100%	264 (28.1%)	216 (27.0%)	48 (34.3%)	0.047
Newly diagnosed Diabetes	100%	28 (3.0%)	19 (2.4%)	9 (6.4%)	0.188
Hypertension	100%	511 (54.5%)	405 (50.8%)	106 (75.7%)	0.002
Current smoking	60%	73 (12.9%)	50 (10.2%)	23 (29.9%)	0.252
**Comorbidities, n (%)**					
Cardiovascular disease	98%	223 (24.1%)	183 (23.1%)	40 (30.3%)	0.784
Atrial fibrillation	99%	77 (8.3%)	56 (7.1%)	21 (15.7%)	0.990
CKD	100%	194 (20.7%)	136 (17.0%)	58 (41.4%)	< 0.001
COPD	98%	63 (6.8%)	46 (5.8%)	17 (12.8%)	0.421
Cancer	98%	90 (9.8%)	66 (8.4%)	24 (18.2%)	0.775
**Symptoms at admission**					
Time from symptoms to hospitalization, days	95%	7.6 ± 4.6	7.7 ± 4.5	6.7 ± 5.5	0.326
Body temperature (°C)	75%	38.0 ± 0.9	38.0 ± 0.9	38.2 ± 0.9	0.420
Cough, %	95%	598 (67.1%)	528 (68.7%)	70 (57.4%)	0.174
Dyspnea, %	96%	537 (59.6%)	441 (57.1%)	96 (75.0%)	0.003
Pneumonia / ILD, %	97%	855 (94.3%)	734 (94.1%)	121 (95.3%)	0.586
GI symptoms, %	94%	237 (26.8%)	204 (26.8%)	33 (27.0%)	0.573
**Medication before hospitalization**					
ACE inhibitors, %	100%	160 (17.1%)	124 (15.6%)	36 (25.7%)	0.163
Angiotensin receptor blockers, %	100%	171 (18.2%)	135 (16.9%)	36 (25.7%)	0.070
Calcium channel blockers, %	100%	158 (16.9%)	125 (15.7%)	33 (23.6%)	0.051
Beta blockers, %	100%	217 (23.1%)	169 (21.2%)	48 (34.3%)	0.053
Anti-platelet agents, %	100%	122 (13.0%)	90 (11.3%)	32 (22.9%)	0.578
Statins, %	100%	108 (11.5%)	81 (10.1%)	27 (19.4%)	0.542
Oral Anticoagulants, %	100%	66 (7.0%)	46 (5.8%)	20 (14.3%)	0.876
Antibiotics, %	100%	484 (51.6%)	431 (54.0%)	53 (37.9%)	0.487
NSAID, %	100%	102 (10.9%)	88 (11.0%)	14 (10.0%)	0.069
**Parameters at admission**					
Systolic blood pressure, mm Hg	99%	129.5 ± 18.7	129.1 ± 17.7	132.2 ± 23.5	0.911
Diastolic blood pressure, mm Hg	99%	79.4 ± 11.8	79.7 ± 11.6	77.8 ± 12.9	0.755
Heart rate, bpm	99%	87.2 ± 14.9	86.8 ± 14.3	89.6 ± 18.1	< 0.001
Respiratory rate /min	88%	21.5 ± 6.7	21.1 ± 6.6	24.0 ± 6.9	< 0.001
Oxygen Saturation, %	99%	93.9 ± 5.3	94.3 ± 4.9	91.5 ± 7.0	< 0.001
Fasting plasma glucose, mmol/l	88%	7.3 ± 3.4	7.1 ± 3.2	8.3 ± 4.4	0.036
HbA1c, mmol/mol	23%	58.0 ± 23.9	61.2 ± 25.6	48.5 ± 14.2	0.111
Serum creatinine, umol/l	100%	96.7 ± 57.3	89.0 ± 28.3	140.4 ± 123.4	< 0.001
eGFR, ml/min/.173 m^2^	100%	72.0 ± 23.3	75.3 ± 21.5	53.6 ± 24.5	< 0.001
White blood cells, cel/µl	87%	6.7 ± 7.0	6.5 ± 7.3	7.8 ± 4.6	0.273
Hematocrit %	98%	40.8 ± 4.9	40.8 ± 4.6	41.0 ± 6.0	0.783
Platelets, el/ul	100%	209.8 ± 84.2	210.9 ± 84.9	203.3 ± 80.5	0.812
C-reactive protein, mg/dl	93%	67.7 ± 75.2	58.7 ± 68.3	120.9 ± 90.6	< 0.001
D-dimer, ug/l	71%	577.4 ± 2182.3	518.1 ± 2242.3	848.7 ± 1867.5	0.878
**Outcomes, n (%)**					
ICU and/or Death	100%	151 (16.1%)	66 (8.3%)	85 (60.7%)	< 0.001
Death	100%	89 (9.5%)	34 (4.3%)	55 (39.3%)	< 0.001
Discharged alive at 30 days	100%	786 (83.7%)	721 (90.2%)	65 (46.4%)	< 0.001
Mean days of hospitalization*	91%	13.9 ± 6.3	13.5 ± 5.9	17.2 ± 8.0	0.005

CKD, chronic kidney disease. COPD, chronic obstructive pulmonary disease. ICU: admission to Intesive Care Units; Pneumonia / ILD: radiological confirmed pneumonia or interstitial lung disease. G.I., gastrointestinal. ACE,angiotensin-converting enzyme. NSAID, non-steroidal anti-inflammatory drugs. eGFR estimated glomerular filtration rate. PaO_2_, partial oxygen pressure. * Among survivors and truncated at 30-days.

### AKI and outcomes

**Figure 1:**
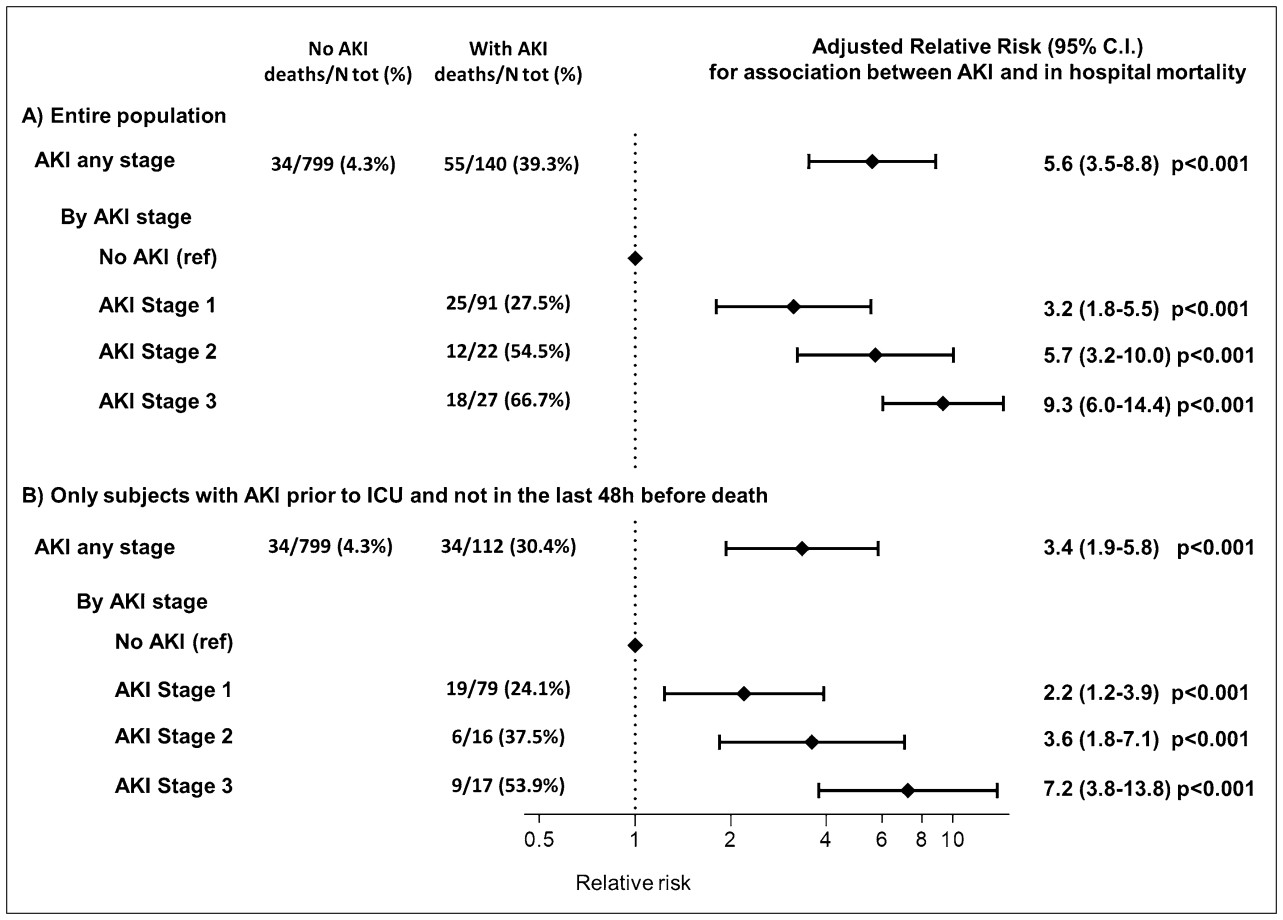
Association between acute kidney injury (AKI) and mortality in the entire population and in sensitivity analyses excluding subjects with AKI onset after deterioration of clinical conditions. Model adjusted by age, sex, clinical center and pre-existing condition associated with both death and AKI (i.e. CKD and diabetes).

**Figure 2:**
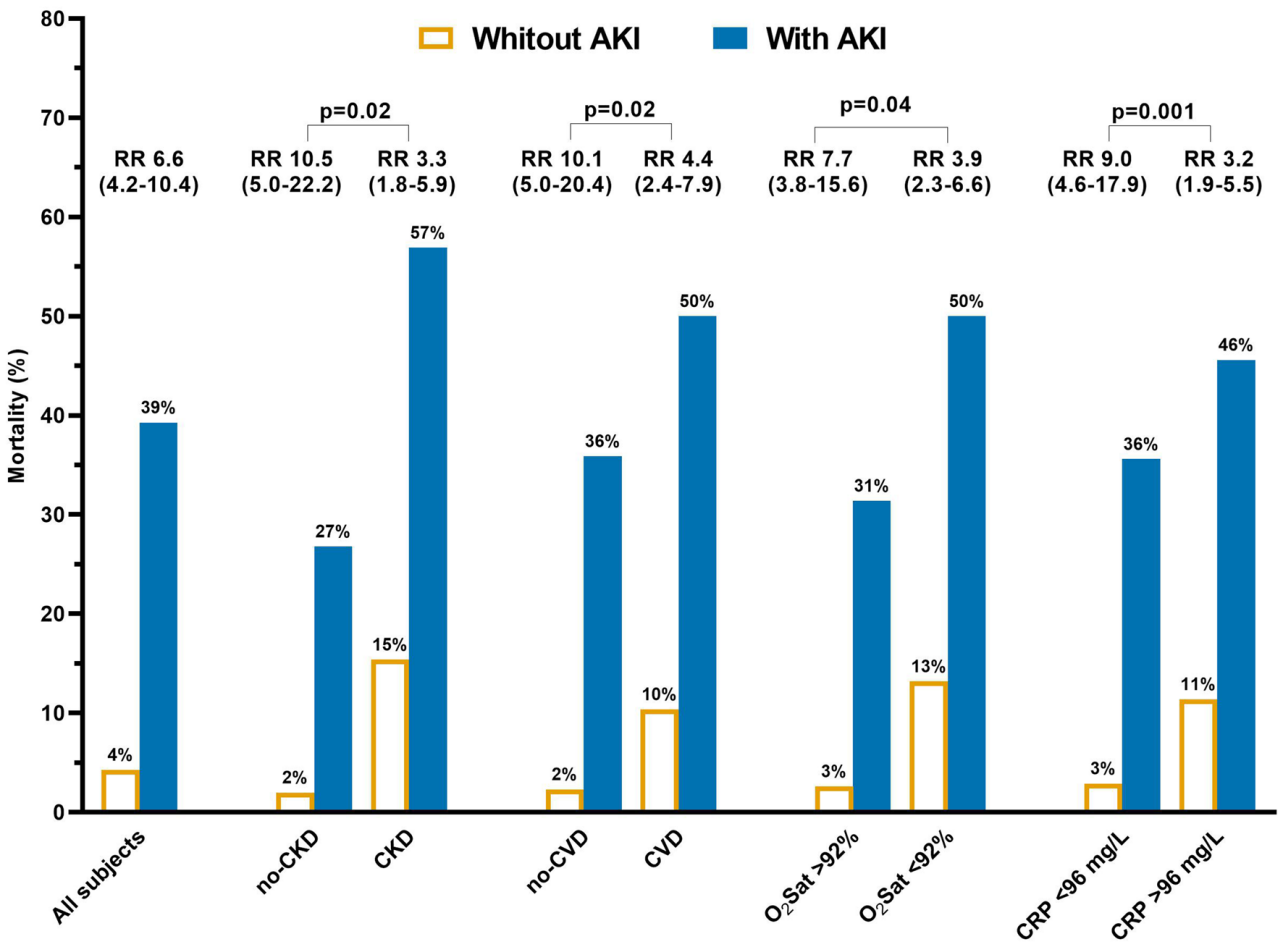
Association between acute kidney injury (AKI) and death according to patients’ characteristics. *Notes*: p for interaction are shown (i.e. testing the differences between the two groups of the association between AKI and death). N.B. Cutoff for oxygen saturation (O_2_ Sat) and C-reactive Protein (CRP) corresponded to top quartile of the population distribution of each variable; analyses adjusted by age, sex and clinical center).

Over a median observation of 14 days (IQR 9–17), 151 patients (16.1%) had a severe course (defined as admission to ICU or death), including 89 patients that died during the hospitalization (11.6%). In age and sex adjusted analyses, presence of diabetes, CKD, dyspnea, shorter duration between symptom onset and hospitalization, presence of gastrointestinal symptoms, and prior use of NSAID were associated with higher mortality (supplementary table S3). In-hospital mortality was much higher among patients with AKI than those without AKI (39.3% vs. 4.3%; unadjusted RR 9.2; 95% C.I. 6.3–13.6; p < 0.001), and increased progressively with increasing AKI stages (AKI stage 1: 27.5%, Stage 2: 54.6%; Stage 3: 66.7%, p for trend < 0.001). Among survivors, AKI was associated with a longer duration of hospitalization (17.2 days Vs 13.5 days, p = 0.005). As shown in Fig. 1 and Table 2, the increased mortality associated with AKI was independent from confounding factors as tested in different models with multiple adjustments and increasing complexity. The association between AKI and mortality was consistent across different baseline clinical characteristics (supplementary figure S1), and the mortality rate of subjects with pre-existing conditions (i.e. CKD or CVD) was largely higher among those also experiencing AKI (Fig. 2). However, the relative impact of AKI on mortality was stronger in subjects without pre-existing CVD or CKD, and in those with oxygen saturation > 92% or low C-reactive protein at admission (all p < 0.05, Fig. 2).

**Table 2:** Association between AKI and mortality risk after multiple adjustments for confounding factors. Note: Models 1 to Model 3 follow an increasing complexity: Model 1: age, sex and clinical center; Model 2 = Model 1 + diabetes and chronic kidney disease; Model 3 = Model 2 + dyspnea, heart rate, respiratory rate, oxygen saturation and C-reactive protein (i.e. those pre-existing conditions and parameters at admission associated both with higher AKI and mortality risk).

	Entire population (n = 939; deaths = 89)		Only those with AKI onset before ICU and at least 48 h before death (n = 911; deaths = 68)	
	R.R. (95% .I.)	P	R.R. (95% .I.)	P
Unadjusted	9.2 (6.3–13.6)	< 0.0001	7.1 (4.6–11.0)	< 0.0001
Model 1	6.6 (4.2–10.4)	< 0.0001	4.1 (2.4–6.9)	< 0.0001
Model 2	5.6 (3.5–8.8)	< 0.0001	3.4 (1.9–5.8)	< 0.0001
Model 3	3.7 (2.2–6.0)	< 0.0001	2.5 (1.4–4.6)	0.0025

Consistently with the higher mortality rate, patients with AKI had also worse in-hospital clinical and laboratory parameters than subjects not developing AKI (i.e., lower oxygen saturation, blood pressure, hematocrit and platelet levels, with higher leukocytosis, inflammatory markers, procalcitonin, and D-dimer levels, as shown in supplementary table S4). Therefore, to dissect whether AKI was preceding worsening clinical condition in COVID-19 patients rather than being a late or concomitant manifestation of a deteriorating clinical situation, we performed the analyses on mortality excluding 28 subjects with AKI occurring after admission to ICU or in the two days preceding death. The association was confirmed, with the onset of AKI being associated with a 240% higher risk of mortality (RR 3.4, 95% C.I. 1.9 to 5.8, p < 0.001, Table 2). Moreover, also AKI stage 1 was consistently associated with a 2.2 to 3.2 higher risk of in-hospital mortality compared to absence of AKI (all p < 0.001, Fig. 1).

Additionally, also among patients with in-hospital worsening of respiratory function requiring invasive or non-invasive ventilation (i.e. 139 patients), the onset of AKI increased the mortality rate significantly and independently from other confounding factors. Indeed subjects with both AKI and worsening of respiratory function and those with worsening of respiratory function without AKI, had an overall mortality of 54% and 32%, respectively (p for difference in all multivariable adjusted models p < 0.001).

### AKI risk factors at admission and during hospital stay

**Figure 3:**
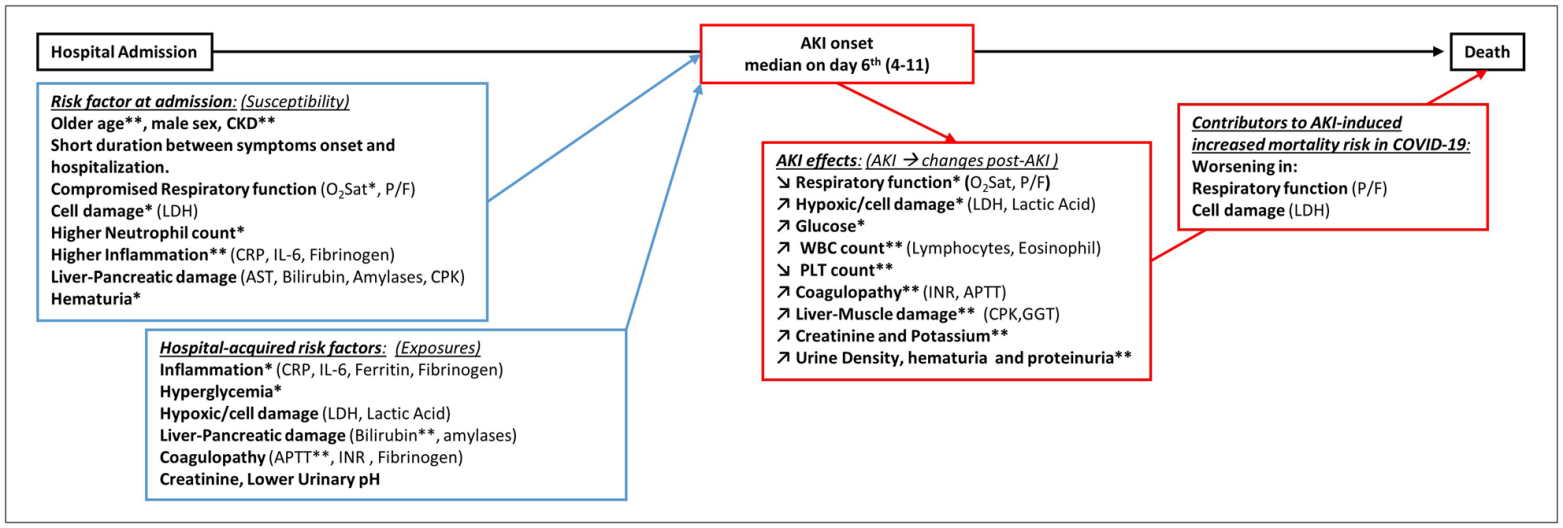
Admission and Hospital-acquired risk factors for AKI onset and contributors of AKI-induced increased mortality risk. *Note*: only patients without AKI at admission and developing AKI before admission to intensive care units are included; *p < 0.001; **p < 0.0001. The other are nominally associated with the outcome (p < 0.05). APTT: activated partial thromboplastin time, AST: aspartate aminotransferase, CKD: chronic kidney disease, CPK: creatine phosphokinase, CRP: C-reactive protein, GGT: gamma-glutamyl transferase, INR: International normalized ratio, LDH: lactate dehydrogenase, O_2_Sat: Oxygen saturation, P/F: Arterial oxygen partial pressure to fractional inspired oxygen ratio.

To evaluate factors associated with AKI onset during hospitalization we analyzed data on 362 patients admitted to the Padova center. As shown in Suppl. Table S5, 72 (19%) subjects developed AKI after admission (median time after 6 days, IQR 4–11), with 51 (71%), 14 (19%), and 7 (10%) having AKI stage 1, 2, and 3 respectively. As summarized in Fig. 3 and Supplementary Table S6, beyond older age, male sex, and presence of CKD, the following risk factors at admission were identified: a worst respiratory function (lower SpO2, P/F), higher neutrophil count, higher inflammatory markers (CRP, IL-6, fibrinogen), presence of liver-pancreatic-muscle damage (higher AST, bilirubin, amylases, LDH, CPK) and hematuria. Moreover, the following parameters were identified as hospital-acquired AKI exposures: increases in inflammatory markers (CRP, Ferritin, Fibrinogen), glucose levels, markers of hypoxic damage and cell death (lactic acid and LDH), liver-pancreatic damage (bilirubin and amylases), presence of coagulopathy (APTT, INR, fibrinogen), and lower urinary pH. For instance, the increase of one SD of CRP (i.e. 55 mg/l) or glucose (i.e. 3.2 mmol/l), were respectively associated with 28% (95% C.I. 7–54) and 33% (95% C.I. 13–56) increased risk of AKI onset.

### Factors influenced by AKI and possible contributors to AKI-induced increased mortality

**Figure 4:**
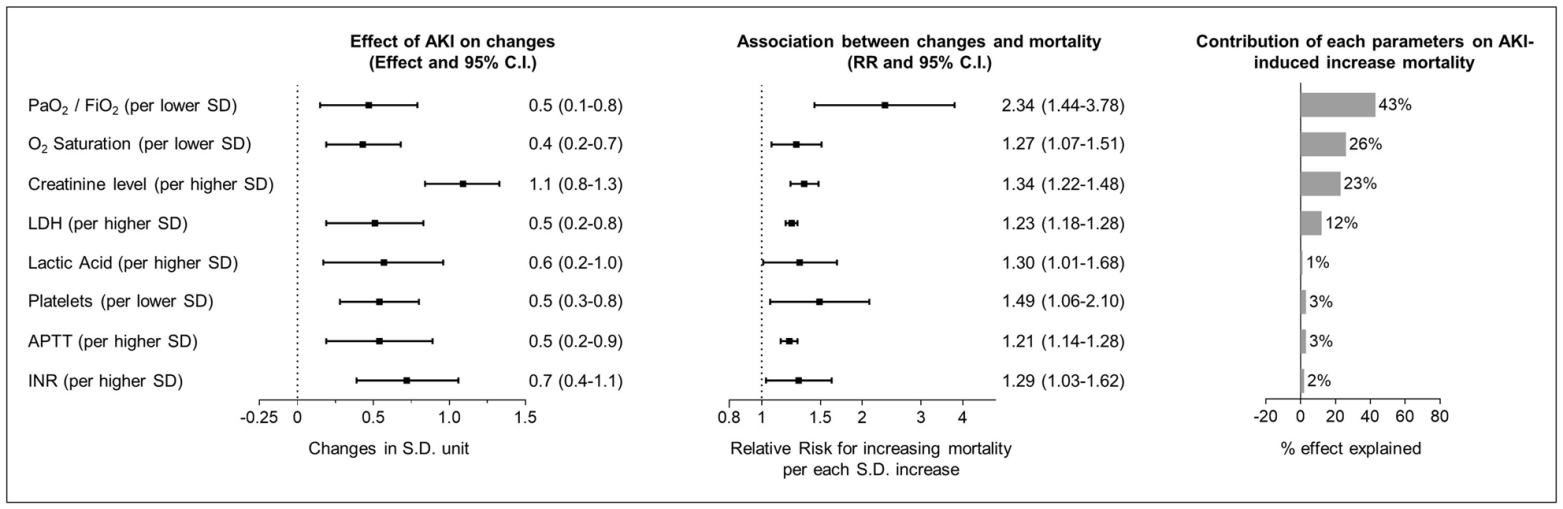
AKI influences on laboratory parameters during hospitalization and possible contributors to AKI-induced increased mortality risk in COVID-19. *Note*: Standard deviations units for each parameters: Oxygen (O2) saturation = 5%; ratio of arterial oxygen partial pressure to fractional inspired oxygen (PaO2/FiO2) = 96 ; Creatinine = 44 umol/l, Lactate dehydrogenase (LDH) = 406 U/L; Lactate Acid 1.4 mmol/l; Platelets = 66 kel/ul; activated partial thromboplastin time (APTT) = 0.31; International normalized ratio (INR) = 0.29.

The onset of AKI after hospital admission was independently associated with a twofold increase in mortality (RR 3.0; 95% C.I. 1.5–6.0, p = 0.001). To identify the possible contributors of such AKI-induced increased mortality, we evaluated factors both worsening after AKI onset and being associated with higher mortality. AKI onset was followed by worsening of respiratory function (SaO2 and P/F), increases in markers of hypoxic damage (lactic acid) and cell death (LDH), higher glucose levels and WBC count, decrease in platelet count and worsening of coagulopathy (elongation on PT and APTT), liver-muscle damage (CPK and GGT), and, as expected, increase in creatinine, potassium levels, urine density and proteinuria (Fig. 3 and detailed in Suppl. Table S7). Of these factors, worsening respiratory function, markers of hypoxic damage and cell death, thrombocytopenia, and alteration of coagulation were associated with higher mortality (Fig. 3 and Suppl. Table S7). However, among these possible contributors, the reduction in platelet count, increases in APTT and INR, did not significantly affect the association between AKI and death. Conversely, the AKI-induced increased mortality was significantly affected by worsening of respiratory function (as detected by a decrease in PaO2/Fio2). Indeed, after adjustment for changes in PaO2/Fio2 the association between AKI and mortality was no longer significant (RR 1.89; 95% C.I. 0.81–4.44) (Fig. 4).

## Discussion

This international multi-center study conveys four key important messages: (1) AKI is a common complication of COVID-19 also in patients hospitalized in non-intensive care units. (2) Beyond the established factors affecting AKI susceptibility (observed at hospital admission), changes in inflammatory markers, glycemia, and indexes of coagulopathy and hypoxic damages represent hospital-acquired exposures increasing the risk of AKI. (3) AKI at any stage, including stage 1, is independently and strongly associated with mortality. (4) AKI-related worsening of respiratory function could be one of the the main driver of the AKI-induced increase in mortality risk.

All these results highlight important and novel clinical insights. Previous studies have reported a highly variable prevalence of AKI among patients hospitalized for COVID-19 (from 4 to 40%)^1,3,9,10^. However, most studies were on severely ill patients, with a large proportion admitted in ICU, and none of them distinguished whether AKI preceded or followed admission to ICU. Our international study adds important information since it was conducted in medical departments, thus providing information on AKI onset in the non-ICU setting. In our cohort, the prevalence of AKI was 14.9% and, according to the KDIGO classification, 65% of patients had AKI stage 1. Considering those of previous studies demonstrating that even a slight serum creatinine increase (i.e. > 0.3 mg/dl in 48 h, corresponding to Stage 1 AKI) may result in medium-long term complications (increased mortality and risk to develop CKD or End-Stage Kidney disease requiring dialysis)^11–13^, this is an important observation with implications for health care systems.

The risk factors for AKI onset in patients with COVID-19 pneumonia are poorly understood, as recently recognized by the consensus report of the 25th Acute Disease Quality Initiative Workgroup on COVID-19 associated AKI^8^. The onset of AKI during hospitalization can be considered as the results from an interplay between patient susceptibility (i.e. risk factors at admission) and many exposures during hospitalization (i.e. hospital-acquired risk factors). In this context, most studies on AKI have identified only risk factors at hospital admission (e.g. older age, male gender, CKD, diabetes, hypertension, worst clinical presentation with higher COVID-19 severity)^4,5,14–16^, but no study aimed to identify possible hospital-acquired risk factors. Conversely, we provide novel information on factors acting as possible in-hospital exposures increasing the risk of AKI onset (e.g. worsening of respiratory function, hypoxic damage, inflammation, hyperglycemia, and coagulopathy). Identification of these elements may guide KDIGO preventive/protective strategies and prioritize resource allocation in times of pandemic with high health care demand^8^. They might improve the identification of subjects at higher risk of AKI during hospitalization, and help physicians to monitor, anticipate, and possibly prevent AKI onset. Although the observational design of this study does not allow establishing any causal relationship, some of these factors might be causally associated with AKI onset. For example, inflammation and hyperglycemia are of particular interest, and further studies are recommended to evaluate whether might be considered as “actionable and modifiable factors” to prevent progression towards AKI.

Acute kidney injury has already been recognized as a complication in patients hospitalized for severe COVID-19 infection associated with increased mortality, length of hospital stay, and healthcare costs^17^. However, only a few studies evaluated the impact of AKI on mortality according to AKI stages, and none distinguished between AKI onset before ICU admission or whether AKI onset followed the deterioration of the clinical condition of patients. Again, our study provides detailed and novel clinical information. Indeed, it reports that the development of mild-to-severe AKI (any stage) was independently associated with markedly increased mortality in patients with Sars-Cov2 infection also in a non-intensive care setting. Most importantly, given the consistency of the results after exclusion of subjects developing AKI after deterioration of general clinical conditions, our study further supports the concept that patients with Sars-Cov2 infection and AKI, die because of AKI and not “simply” with AKI. Remarkably, while the mortality rate was the highest among those subjects developing AKI and having concomitant pre-existing conditions (e.g. CKD and/or CVD), the relative impact of AKI on mortality was even more clearly emerging among patients without CKD or CVD. In these subjects, where mortality was overall lower, AKI onset was associated with almost tenfold increase in mortality rate as compared to subjects without AKI.

Finally, we identified factors both possibly influenced by AKI (i.e. with significant changes after AKI onset), and associated with higher mortality, as possible contributors of the AKI-associated higher mortality. These factors were worsening of respiratory function, hypoxic damage and cellular deaths (higher lactic acid and LDH), thrombocytopenia, coagulopathy (higher INR and APTT), and creatinine levels. However, we found the AKI-associated reduction in platelet count or increases in APTT and INR, did not affect the association between AKI and death. Therefore, these changes were more likely epiphenomena to AKI onset and not contributors to its effect on mortality. On the other hand, our data suggest that the AKI-induced increased mortality risk was mainly attributable to the worsening of respiratory function. In other words, our data showed that a worst respiratory function at admission was a risk factor for AKI onset, and that beyond such association, AKI onset antecede a further deterioration in the respiratory function that was found to explain about 26–43% of the AKI-induced increase in mortality. Is it important to highlight that the retrospective observational design of our study warrant caution before implying the causality of these association. However, in line with this, we observed that AKI was followed by a significant increase in LDH and that such increase was independently associated with higher mortality. While LDH is more likely to be a marker and not an effector or mediator of AKI-induced mortality (it only minimally affect the association between AKI and mortality), it has been previously reported as an important biomarker for the activity and severity of interstitial lung disease^18,19^, and as a prognostic factor in COVID-19^20,21^. Therefore, this suggest that AKI onset could also have a direct effect of worsening of interstitial lung disease and therefore on mortality in patients with COVID-19 pneumonia. Such hypothesis will require further studies to be confirmed and to dissect the mechanisms involved (e.g. increased lung congestion, defective viral clearance [that might lead to more extend viral dissemination and higher cell deaths] or increased inflammation).

Overall, this study shows how AKI (at any stage) had an important impact on overall mortality in all subgroups of patients hospitalized with COVID-19. Indeed, AKI increased mortality among those patients without pre-existing concomitant conditions or among those with preserved respiratory function at admission, but also among those patients being admitted with severe clinical conditions. For instance, patients with low oxygen saturation or those that required in-hospital invasive or non-invasive ventilation had a much higher mortality if they also had AKI as compared to those who did not had AKI. Therefore, while all patients with low oxygen saturation at admission had “per se” a tendency to get in-hospital worsening of respiratory function and therefore of an higher COVID-19 mortality risk, those patients developing also AKI are those who should be considered at the highest mortality risk (that is likely driven, in part, by an AKI-associated further decline in respiratory function).

Our study should be interpreted in the context of several limitations. First, real-world routinely accumulated clinical data can be affected by missing data, however, we applied extensive multiple imputation techniques to avoid excluding patients from the analyses. Second, this study combined patients hospitalized from different countries and with different demographic backgrounds, hence with different clinical characteristics. On one side this approach increases generalizability of our findings, but on the others sides this approach also introduces variability in measured and unmeasured factors across centers. While the measured differences were accounted for in the analyses, ideally, to further dissect the risk factors and outcomes of AKI, further studies with a more homogeneous population might be warranted. Third, the retrospective design did not allow the collection of ad-hoc important variables such as urinary outputs, changes in body weight, and more advanced serum and urinary inflammatory markers that are not routinely analyzed or recorded. We were unable to include medications as possible exposures, since detailed information with changes in treatments during hospitalization was not available. Therefore, it is important to recognize that other risk factors (e.g. exposures) might be present and future studies should consider them.

In conclusion, AKI is a common complication of Sars-Cov-2 infection in medical departments (outside ICU), with a high prevalence of AKI stage 1. AKI at any stage is an independent risk factor for mortality in a non-intensive care setting. The worsening of respiratory function following AKI is a possible main contributor to AKI-induced increased in mortality. Early identification of hospital-acquired risk factors for AKI (worsening of respiratory function, hypoxic damage and cellular death, inflammation, hyperglycemia, and coagulopathy) might help developing new strategies to mitigate AKI onset and impact COVID-19 mortality, as well as long-term consequences of AKI in COVID-19 patients.

## Methods

### Study design

The study was carried out collecting retrospective anonymized patient data from electronic medical records at participating centers. The protocol conforms to the ethical guidelines of the 1975 Declaration of Helsinki. In agreement with national regulation on retrospective studies, the protocol was notified to the local ethical committees (no. 0048697 at the University Hospital of Padova, Italy and no. 6, 30 April 2020 at committee of Endocrinology Research Center, Moscow, Russia), and the need for the patient's informed consent was waived.

### Data collection

#### Italy-Covid population

 We retrieved data on all consecutive patients hospitalized during the first Sars-CoV-2 outbreak in Northern Italy (between February 21st and April 20th, 2020), at the Medical Department of the University Hospital of Padova (Internal Medicine, Infectious Disease and Pneumology). We screened records of all patients admitted to the hospital with a positive PCR test for SARS-CoV-2 on upper or lower airway samples, excluding those initially admitted in ICU.

#### Russia-Covid population

 Patients from two different hospitals were included. All consecutive patients hospitalized with COVID-19 between April 5th and May 5th 2020, at the Endocrinology Research Center, Moscow, Russia, and randomly chosen 339 of 1207 patients hospitalized with COVID-19 between April 13th and August 2nd 2020, from the Tareev Clinic of Internal Diseases, Sechenov University, Moscow, Russia. The COVID-19 was confirmed with a positive PCR test for SARS-CoV-2 and/or by typical CT findings. Overall, 293 patients had a positive PCR test while the remaining had COVID19 diagnosis based on clinical and CT findings (i.e. the SARS-CoV-2 pneumonia defined as acute respiratory infection with typical CT findings [bilateral multilobar ground-glass opacification with a peripheral or posterior distribution, or multifocal consolidative opacities superimposed on ground-glass opacification] and no other obvious etiology, with a CO-RADS scale of 4 or more)^22^.

The following parameters were collected and analyzed in all patients: age, sex, concomitant cardiovascular risk factors (smoke, hypertension), diabetes, CKD, other comorbidities (chronic obstructive pulmonary disease, history of cancer, cardiovascular disease, atrial fibrillation), presence or absence of COVID-19 related pneumonia or interstitial lung disease (ILD), ongoing therapies before hospitalization were evaluated. Community-acquired AKI was documented by increased creatinine values at admission compared to historical values or values recorded until 48 h before admission. Pre-existing CKD was evaluated according to medical diagnosis at admission and GFR estimation. Pre-existing diabetes and newly diagnosed diabetes was defined as previously described^2^. Vital signs (blood pressure, heart rate, respiratory rate, peripheral oxygen saturation) and information on symptoms of COVID-19 (collected upon admission): time from onset of symptoms to hospitalization; the presence of fever, cough, dyspnea, and gastrointestinal symptoms) were collected. Essential laboratory exams at admission included: fasting plasma glucose, HbA1c, serum creatinine to estimate glomerular filtration rate (eGFR) using the CKD-EPI equation, white blood cell count (WBC), Hematocrit, Platelet count, C-reactive protein (CRP), and D-dimer. The same parameters were also measured during the entire hospital stay, and the worst value was considered (admission or any time during hospitalization).

### AKI definition

AKI stage was defined according to KDIGO criteria^23^ based on changes in creatinine levels criteria (urine volume criteria were not applied since such information were not available). Creatinine was evaluated every 48–72 h allowing identification of the worst AKI stage throughout the hospital stay.

### Outcomes

We evaluated the cumulative incidence of AKI throughout the hospitalization period and compared it with the primary outcome of the study that was 30-day in-hospital mortality.

Development of AKI at least 48 h after admission and before admission to ICU was considered as a secondary outcome in the analyses aimed at identifying admission and hospital-acquired factors associated with the onset of AKI.

### Risk factors at admission and Hospital-acquired risk factors for AKI onset

To identify risk factors at admission (AKI susceptibility) or during hospitalization (AKI exposure) associated with the onset of AKI during hospitalization, we analyzed in detail a subgroup of patients admitted to the University Hospital of Padova with AKI onset at least 48 h after admission and before eventual ICU admission. For these patients, complete data on multiple laboratory variables was available throughout the hospitalization stay.

We evaluated as risk factors at admission (susceptibility): age, sex, comorbidities (diabetes, CKD, chronic obstructive pulmonary disease, history of cancer, cardiovascular disease, atrial fibrillation), symptoms at admission, medication before hospitalization, blood laboratory results (creatinine, potassium, hematocrit, Hemoglobin, Platelets, White blood cells, Lymphocytes, Neutrophils, Eosinophils, Monocytes, C-reactive protein, Ferritin, Fibrinogen, Plasma glucose, LDH,\ Lactic Acid, CPK, Troponin I, AST, ALT, GGT, Bilirubin, Amylases, APTT, INR, D-dimer) and urine biochemical results (Urine density, Ph, Hb, Protein, Glucose, and Ketones). The changes from baseline in these parameters (the first available within the first 2 days of hospitalization) to the worst value recorder during hospitalization and before AKI onset (up to the day before the AKI onset) were evaluated as possible Hospital-acquired risk factors for AKI onset (exposures).

The same variables were tested as possible factors contributing to AKI-induced increase in mortality risk. Specifically, we evaluated the changes from the first value available from the day of AKI onset to the worst value recorder after AKI onset to identify those factors influenced by AKI and associated with death (worst value throughout hospitalization was considered).

### Statistical analysis

Continuous variables are reported as mean ± standard deviation (S.D.) or median (inter-quartile range), while categorical variables have been reported with percentages.

We tested the association of variables of interest with AKI occurrence and death with robust-error-variance Poisson regression models^24^, included sex, age, and clinical center as covariates (Model 1). To account for other confounding factors, the effect of AKI on mortality was tested in different multivariable-adjusted analyses with increasing complexity: including, on top of Model 1, those pre-existing conditions (Model 2) and parameters at admission (Model 3) associated both with AKI and mortality risk. The association between AKI and death was evaluated both as a dichotomous variable and as an ordinal categorical variable testing the contribution of each AKI stage as compared to subjects without AKI. The different effect of AKI on mortality according to baseline patient’s characteristics, was tested including an interaction term in the Model 1. We evaluated the association between AKI and severity of COVID-19 (defined by the admission to ICU or death) by robust-error-variance Poisson regression and between AKI and length of hospital stay (among survivors) employing linear regression (with same covariates as in Model 1). Poisson regression models were tested for overdispersion with analyses of goodness-of-fit. Sensitivity analyses were further conducted in a subgroup of subjects including only those with AKI onset before admission to ICU and at least three days before death. To account for the differences between subjects hospitalized in Italy and Russia (Supplementary table S1), all the models included a covariate defining the clinical center.

To test the multivariable adjusted models, a full dataset of variables was needed. Thus, missing data were handled using multiple imputation (MI). MI was performed, assuming a missing at random (MAR) pattern, with a fully conditional specification (FCS) algorithm^25^ obtaining ten imputed datasets including only covariates with less than 60% of missing values. Outcome variables and main variables of interest (i.e. AKI) were not imputed. Outcome analyses were performed on each imputed dataset, and the pooled estimated effects are presented^26^.

Additional analyses were performed in the group of subjects with onset of AKI during stay in the non-ICU setting. To identify factors (at admission or hospital-acquired) associated with the onset of AKI, we used the same model previously described (Model 1). To test whether AKI onset was associated with changes during hospitalization in variables of interest, we performed linear regression analyses including age, sex, and value at baseline (or at AKI onset) as covariates. To allow a meaningful interpretation and comparison of the effect between different parameters the effects were rescaled to the Standard Deviation of each variable. Finally, we tested whether the association between AKI and mortality was partially mediated by factors both influenced by AKI and associated with higher mortality, as previously described^2^. Briefly, we evaluated whether the association between AKI and mortality was affected, and in which proportion, after including in the model the changes in the parameters affected by AKI-onset. Statistical analyses were conducted with a significance threshold of p < 0.05 and were done in SAS version 9.4 (TS1M4), GraphPad PRISM v8.3.0.

### IRB approval

In agreement with national regulations on retrospective studies, the protocol was notified to the local ethical committees (no. 0048697 at the University Hospital of Padova, Italy and no. 6, 30 April 2020 at committee of Endocrinology Research Center, Moscow, Russia), and the need for the patient's informed consent was waived.

## Supplementary Information

Supplementary Information.

## Data Availability

Original data are available from the corresponding author at a reasonable request.
