# Supplementary material for: In hospital risk factors for acute kidney injury and its burden in patients with Sars-Cov-2 infection: a longitudinal multinational study
Source: Sci Rep. 2022 Mar 2;12:3474. doi: 10.1038/s41598-022-07490-z (PMC8891366; doi:10.1038/s41598-022-07490-z)
Supplement: Supplementary file 1 — Supplementary Information. [file 41598_2022_7490_MOESM1_ESM.docx]

**Supplementary tables and figures of Manuscript: “In hospital risk factors for acute kidney injury and its burden in patients with Sars-Cov-2 infection: a longitudinal multinational study”**

***Authors:*** Mario Luca Morieri, Claudio Ronco, Angelo Avogaro, Filippo Farnia, Marina Shestakova, Natalya Zaytseva, Natalya Mokrysheva, Larisa Akulkina, Anastasia Shepalina, Michail Brovko, Sergey Moiseev, Lucia Russo, Sara Mazzocut, Andrea Vianello, Anna Maria Cattellan, Monica Vedovato, Gian Paolo Fadini, Roberto Vettor, Paola Fioretto

**Table S1. Characteristics of COVID-19 patients according to Clinical centers**. Data presented as mean (S.D.) or as percentage. In the column reporting data of all patients, data availability is also shown. P value is refers to differences across the clinical centers.

|  | Padua  N=413 | | Moscow_S  N=189 | | Moscow_T  N=337 | | P value |
| --- | --- | --- | --- | --- | --- | --- | --- |
|  | Available | Value |  | Value |  | Value |  |
| **Age, years** | 100% | 64.9 ± 15.4 | 100% | 60.6 ± 16.0 | 100% | 59.0 ± 14.8 | <0.001 |
| **Sex male, %** | 100% | 245 (59.3%) | 100% | 98 (51.9%) | 100% | 154 (45.7%) | <0.001 |
| **Concomitant Risk Factors, n (%)** |  |  |  |  |  |  |  |
| **Known Diabetes** | 100% | 86 (20.8%) | 100% | 42 (22.2%) | 100% | 136 (40.4%) | <0.001 |
| **Hypertension** | 100% | 212 (51.3%) | 99% | 91 (48.7%) | 100% | 208 (61.7%) | <0.001 |
| **Current smoking** | 46% | 53 (27.7%) | 20% | 8 (21.1%) | 100% | 12 (3.6%) | <0.001 |
| **Comorbidities, n (%)** |  |  |  |  |  |  |  |
| **Cardiovascular disease** | 97% | 72 (18.0%) | 99% | 26 (13.8%) | 100% | 125 (37.1%) | <0.001 |
| **Atrial fibrillation** | 97% | 44 (11.0%) | 99% | 10 (5.3%) | 100% | 23 (6.8%) | 0.3 |
| **CKD** | 100% | 71 (17.2%) | 99% | 38 (20.2%) | 100% | 85 (25.2%) | <0.001 |
| **COPD** | 97% | 29 (7.3%) | 99% | 20 (10.6%) | 100% | 14 (4.2%) | 0.5 |
| **Cancer** | 96% | 66 (16.6%) | 98% | 9 (4.8%) | 100% | 15 (4.5%) | <0.001 |
| **Symptoms at admission** |  |  |  |  |  |  |  |
| **Days from symptoms to hospitalization** | 88% | 7.0 ± 4.7 | 99% | 8.4 ± 4.0 | 100% | 7.7 ± 4.9 | 0.002 |
| **Body temperature (°C)** | 44% | 38.5 ± 0.6 | 100% | 38.2 ± 0.9 | 100% | 37.6 ± 0.8 | <0.001 |
| **Cough, %** | 89% | 234 (63.9%) | 99% | 137 (72.9%) | 100% | 227 (67.4%) | 0.3 |
| **Dyspnea, %** | 91% | 232 (61.9%) | 100% | 112 (59.3%) | 100% | 193 (57.3%) | 0.5 |
| **Pneumonia / ILD, %** | 92% | 332 (87.1%) | 100% | 189 (100%) | 100% | 334 (99.1%) | <0.001 |
| **GI symptoms, %** | 87% | 102 (28.5%) | 99% | 49 (26.1%) | 100% | 86 (25.5%) | 0.2 |
| **Medication before hospitalization** |  |  |  |  |  |  |  |
| **ACE inhibitors, %** | 100% | 71 (17.2%) | 98% | 39 (21.0%) | 100% | 50 (14.8%) | 0.2 |
| **ARBs, %** | 100% | 73 (17.7%) | 99% | 32 (17.1%) | 100% | 66 (19.6%) | 0.1 |
| **Calcium channel blockers, %** | 100% | 61 (14.8%) | 99% | 35 (18.7%) | 100% | 62 (18.4%) | 0.004 |
| **Beta blockers, %** | 100% | 87 (21.1%) | 100% | 47 (24.9%) | 100% | 83 (24.6%) | 0.002 |
| **Anti-platelet agents, %** | 100% | 73 (17.7%) | 100% | 17 (9.0%) | 100% | 32 (9.5%) | 0.03 |
| **Statins, %** | 100% | 81 (19.6%) | 99% | 12 (6.4%) | 100% | 15 (4.5%) | <0.001 |
| **Oral Anticoagulants, %** | 100% | 45 (10.9%) | 99% | 7 (3.7%) | 100% | 14 (4.2%) | 0.02 |
| **Antibiotics, %** | 100% | 135 (32.7%) | 99% | 169 (89.9%) | 100% | 180 (53.4%) | <0.001 |
| **NSAID, %** | 100% | 24 (5.8%) | 99% | 29 (15.4%) | 100% | 49 (14.5%) | <0.001 |
| **Parameters at admission** |  |  |  |  |  |  |  |
| **Systolic BP, mm Hg** | 98% | 130.1 ± 20.0 | 100% | 131.9 ± 19.5 | 100% | 127.5 ± 16.3 | 0.51 |
| **Diastolic BP, mm Hg** | 98% | 76.8 ± 11.8 | 100% | 80.9 ± 13.0 | 100% | 81.7 ± 10.5 | <0.001 |
| **Heart rate, bpm** | 97% | 86.6 ± 15.8 | 100% | 87.8 ± 15.0 | 100% | 87.6 ± 13.8 | 0.92 |
| **Respiratory rate /min** | 74% | 20.8 ± 6.2 | 100% | 23.4 ± 5.9 | 100% | 21.1 ± 7.5 | <0.001 |
| **Oxygen saturation, %** | 98% | 94.6 ± 4.5 | 100% | 91.9 ± 5.2 | 100% | 94.2 ± 6.1 | <0.001 |
| **Fasting plasma glucose, mmol/l** | 76% | 7.2 ± 3.5 | 95% | 7.4 ± 3.5 | 99% | 7.2 ± 3.4 | 0.26 |
| **HbA1c, mmol/mol** | 34% | 50.0 ± 17.5 | 16% | 69.5 ± 18.4 | 12% | 76.3 ± 31.7 | <0.001 |
| **Serum creatinine, umol/l** | 100% | 95.2 ± 75.4 | 98% | 89.3 ± 42.6 | 100% | 102.7 ± 33.0 | 0.21 |
| **White blood cells, kel/µl** | 70% | 6.7 ± 3.5 | 100% | 8.1 ± 13.3 | 100% | 5.9 ± 2.9 | 0.25 |
| **Hematocrit %** | 96% | 41.7 ± 7.0 | 100% | 40.4 ± 4.8 | 99% | 40.5 ± 4.9 | 0.01 |
| **Platelets, el/ul** | 100% | 209.4 ± 84.6 | 100% | 224.1 ± 89.3 | 100% | 202.2 ± 79.9 | 0.47 |
| **C-reactive protein, mg/dl** | 84% | 84.9 ± 80.0 | 100% | 35.9 ± 64.5 | 100% | 67.8 ± 69.9 | <0.001 |
| **D-dimer, ug/l** | 90% | 767.3 ± 2827 | 81% | 473.8 ± 870.2 | 41% | 183.2 ± 549 | 0.08 |
| **Outcomes and worst in-hospital parameters, n (%)** | 100% |  | 100% |  | 100% |  |  |
| **AKI** |  | 107 (25.9%) |  | 12 (6.3%) |  | 21 (6.2%) | <0.001 |
| **Worst in-hospital Oxygen saturation** |  | 91.6 ± 6.0 |  | 90.1 ± 7.9 |  | 92.2 ± 5.2 | 0.60 |
| **Intubation** |  | 53 (12.8%) |  | 16 (8.5%) |  | 26 (7.7%) | 0.46 |
| **Death** |  | 48 (11.6%) |  | 16 (8.5%) |  | 25 (7.4%) | 0.86 |

CKD, chronic kidney disease. COPD, chronic obstructive pulmonary disease. ILD, interstitial lung disease. G.I., gastrointestinal. ACE, angiotensin converting enzyme. NSAID, non-steroidal anti-inflammatory drugs. HDL, high density lipoprotein. LDL, low density lipoprotein. eGFR, estimated glomerular filtration rate. PaO_2_, partial oxygen pressure.

**Supplementary Table S2. Factors independently associated with AKI occurrence during hospitalization.** Model 1 including all by pre-existing condition and medication significantly associated with AKI (as shown in table 1). Model 2: Model 1 variable + including all clinical significantly characteristics at admission associated with AKI (all models are also adjusted by clinical center).

| **Model 1** | **RR (95% C.I.)** | **P** |  | **Model 2** | **RR (95% C.I.)** | **P** |
| --- | --- | --- | --- | --- | --- | --- |
| **Variable** |  |  |  | **Variable** |  |  |
| **Age (years)** | 1.02 (1.01-1.04) | **0.001** |  | **Age (years)** | 1.02 (1.01-1.03) | **<0.0001** |
| **Sex Male** | 1.75 (1.29-2.38) | **<0.001** |  | **Sex Male** | 1.50 (1.11-2.03) | **0.0088** |
| **Diabetes** | 1.34 (1.00-1.80) | **0.048** |  | **Diabetes** | 1.41 (1.05-1.88) | **0.0208** |
| **Hypertension** | 1.55 (1.02-2.36) | **0.042** |  | **Hypertension** | 1.39 (0.93-2.08) | 0.1081 |
| **Chronic Kidney Disease** | 1.98 (1.44-2.73) | **<0.001** |  | **Chronic Kidney Disease** | 1.46 (1.05-2.01) | **0.0225** |
|  |  |  |  | **Dyspnea** | 1.42 (0.98-2.05) | 0.0616 |
|  |  |  |  | **Heart Rate (bpm)** | 1.01 (1.00-1.02) | 0.0966 |
|  |  |  |  | **Respiratory rate (Breath pm)** | 0.99 (0.97-1.01) | 0.4598 |
|  |  |  |  | **Oxygen Saturation (%)** | 0.96 (0.94-0.98) | **0.0006** |
|  |  |  |  | **C-reactive protein (*50 mg/L)** | 1.13 (1.06-1.21) | **0.0002** |
|  |  |  |  | **Creatinine (*10 umol/L)** | 1.03 (1.02-1.04) | **<0.0001** |

**Supplementary Table S3. Characteristics of COVID-19 patients stratified by survival**. Data presented as mean (S.D.) or as percentage. In the column reporting data of all patients, data availability is also shown.

|  | All patients N=939 | Alive  N=850 | Deceased  N=89 | P (MVA1) |
| --- | --- | --- | --- | --- |
|  | Value | Value | Value |  |
| **Age, years** | 62.0 ± 15.5 | 60.3 ± 14.9 | 77.9 ± 11.7 | <0.001 |
| **Sex male, %** | 497 (52.9%) | 446 (52.5%) | 51 (57.3%) | 0.041 |
| **Concomitant Risk Factors, n (%)** |  |  |  |  |
| **Diabetes (Known+New)** | 292 (31.1%) | 248 (29.2%) | 44 (49.4%) | 0.002 |
| **Known Diabetes** | 264 (28.1%) | 223 (26.2%) | 41 (46.1%) | 0.001 |
| **Newly diagnosed Diabetes** | 28 (3.0%) | 25 (2.9%) | 3 (3.4%) | 0.934 |
| **Hypertension** | 511 (54.5%) | 440 (51.9%) | 71 (79.8%) | 0.322 |
| **Current smoking** | 73 (12.9%) | 65 (12.5%) | 8 (16.7%) | 0.671 |
| **Comorbidities, n (%)** |  |  |  |  |
| **Cardiovascular disease** | 223 (24.1%) | 184 (22.0%) | 39 (45.3%) | 0.230 |
| **Atrial fibrillation** | 77 (8.3%) | 53 (6.3%) | 24 (28.2%) | 0.053 |
| **CKD** | 194 (20.7%) | 140 (16.5%) | 54 (60.7%) | <0.001 |
| **COPD** | 63 (6.8%) | 49 (5.9%) | 14 (16.1%) | 0.364 |
| **Cancer** | 90 (9.8%) | 72 (8.6%) | 18 (20.9%) | 0.135 |
| **Symptoms at admission** |  |  |  |  |
| **Symptoms to hospitalization, days** | 7.6 ± 4.6 | 7.7 ± 4.6 | 5.9 ± 4.7 | 0.040 |
| **Body temperature (°C)** | 38.0 ± 0.9 | 38.0 ± 0.9 | 38.1 ± 0.9 | 0.077 |
| **Cough, %** | 598 (67.1%) | 552 (67.8%) | 46 (59.7%) | 0.999 |
| **Dyspnea, %** | 537 (59.6%) | 474 (58.2%) | 63 (73.3%) | 0.038 |
| **Pneumonia / ILD, %** | 666 (92.8%) | 600 (92.2%) | 66 (98.5%) | 0.360 |
| **GI symptoms, %** | 237 (26.8%) | 227 (28.2%) | 10 (12.8%) | 0.048 |
| **Medication before hospitalization** |  |  |  |  |
| **ACE inhibitors, %** | 160 (17.1%) | 139 (16.4%) | 21 (23.6%) | 0.615 |
| **Angiotensin receptor blockers, %** | 171 (18.2%) | 150 (17.7%) | 21 (23.6%) | 0.635 |
| **Calcium channel blockers, %** | 158 (16.9%) | 139 (16.4%) | 19 (21.3%) | 0.878 |
| **Beta blockers, %** | 217 (23.1%) | 182 (21.4%) | 35 (39.3%) | 0.187 |
| **Anti-platelet agents, %** | 122 (13.0%) | 103 (12.1%) | 19 (21.3%) | 0.816 |
| **Statins, %** | 108 (11.5%) | 94 (11.1%) | 14 (15.9%) | 0.374 |
| **Oral Anticoagulants, %** | 66 (7.0%) | 51 (6.0%) | 15 (16.9%) | 0.900 |
| **Antibiotics, %** | 484 (51.6%) | 445 (52.4%) | 39 (43.8%) | 0.812 |
| **NSAID, %** | 102 (10.9%) | 86 (10.1%) | 16 (18.0%) | <0.001 |
| **Parameters at admission** |  |  |  |  |
| **Systolic blood pressure, mm Hg** | 129.5 ± 18.7 | 129.4 ± 18.2 | 131.3 ± 23.4 | 0.805 |
| **Diastolic blood pressure, mm Hg** | 79.4 ± 11.8 | 79.8 ± 11.6 | 76.3 ± 13.0 | 0.355 |
| **Heart rate, bpm** | 87.2 ± 14.9 | 86.7 ± 14.7 | 92.0 ± 16.2 | <0.001 |
| **Respiratory rate /min** | 21.5 ± 6.7 | 21.2 ± 6.7 | 24.7 ± 6.4 | <0.001 |
| **Oxygen saturation, %** | 93.9 ± 5.3 | 94.4 ± 4.7 | 89.2 ± 8.3 | <0.001 |
| **Fasting plasma glucose, mmol/l** | 7.3 ± 3.4 | 7.1 ± 3.2 | 8.9 ± 4.6 | 0.001 |
| **HbA1c, mmol/mol** | 58.0 ± 23.9 | 58.6 ± 24.5 | 51.8 ± 16.2 | 0.806 |
| **Serum creatinine, umol/l** | 96.7 ± 57.3 | 92.7 ± 54.1 | 135.4 ± 71.2 | <0.001 |
| **eGFR, ml/min/.173 m^2^** | 72.0 ± 23.3 | 74.6 ± 21.8 | 48.0 ± 23.3 | <0.001 |
| **White blood cells, kel/µl** | 6.7 ± 7.0 | 6.6 ± 7.2 | 8.0 ± 5.1 | 0.511 |
| **Hematocrit %** | 40.8 ± 4.9 | 41.0 ± 4.6 | 39.4 ± 6.7 | 0.328 |
| **Platelets, el/ul** | 209.8 ± 84.2 | 211.1 ± 84.5 | 197.2 ± 81.4 | 0.407 |
| **C-reactive protein, mg/dl** | 67.7 ± 75.2 | 61.2 ± 69.6 | 127.8 ± 95.6 | <0.001 |
| **D-dimer, ug/l** | 577.4 ± 2182 | 493.6 ± 2141 | 1300 ± 2410 | 0.079 |

CKD, chronic kidney disease. COPD, chronic obstructive pulmonary disease. ILD, interstitial lung disease. G.I., gastrointestinal. ACE, angiotensin converting enzyme. NSAID, non-steroidal anti-inflammatory drugs. eGFR, estimated glomerular filtration rate. PaO_2_, partial oxygen pressure. IL-6, interleukin-6.

**Suppl Table S4. In-Hospital parameters, treatments, and outcomes according to AKI status**. Note: here we show the clinical-laboratory or parameters evaluated at their worst value measured during the entire hospital stay (regardless of whether it was at admission or during any other time of hospitalization) and in-hospital treatments.

|  | All patients  N=939 | | No AKI  N=798 | AKI  N=140 | P (MVA1) |
| --- | --- | --- | --- | --- | --- |
| **Worst in-hospital parameters** | Available | Value | Value | Value |  |
| **Systolic blood pressure, mm Hg** | 100% | 121.6 ± 31.1 | 124.6 ± 28.6 | 103.8 ± 38.1 | <0.001 |
| **Diastolic blood pressure, mm Hg** | 100% | 74.7 ± 20.5 | 76.9 ± 19.0 | 62.3 ± 24.3 | <0.001 |
| **Heart rate, bpm** | 99% | 91.8 ± 22.0 | 91.5 ± 19.3 | 93.7 ± 33.6 | 0.706 |
| **Respiratory rate / min** | 92% | 23.0 ± 8.7 | 22.5 ± 8.1 | 25.7 ± 11.2 | 0.155 |
| **Oxygen saturation, %** | 100% | 88.8 ± 16.4 | 90.2 ± 14.4 | 81.3 ± 23.5 | <0.001 |
| **White blood cells, kel/µl** | 87% | 7 (5-11) | 7 (5-9) | 14 (8-19) | 0.029 |
| **Hematocrit %** | 98% | 38 (34-41) | 38 (35-41) | 34 (28-38) | <0.001 |
| **Platelets, kel/**$\boldsymbol{\mu}$**l** | 100% | 187 (142-257) | 194 (149-268) | 150 (114-194) | <0.001 |
| **C-reactive protein, mg/dl** | 97% | 105.3 ± 87.9 | 92.4 ± 78.4 | 179.4 ± 102.2 | <0.001 |
| **IL-6, pg/ml** | 36% | 275.1 ± 961.9 | 132.0 ± 428.9 | 819.3 ± 1845.9 | <0.001 |
| **Pro-calcitonin, ug/l** | 36% | 2.0 ± 10.7 | 0.7 ± 6.3 | 5.9 ± 17.9 | <0.001 |
| **D-dimer, µg/l** | 81% | 1649.1 ± 5499 | 1084.7 ± 3420 | 4309.5 ± 10477 | <0.001 |
| **COVID-19 therapies, n (%)** |  | |  |  |  |
| **Oxygen** | 100% | 603 (64.2%) | 474 (59.3%) | 129 (92.1%) | <0.001 |
| **Non-invasive ventilation** | 100% | 101 (10.8%) | 51 (6.4%) | 50 (35.7%) | <0.001 |
| **Invasive ventilation** | 100% | 95 (10.1%) | 38 (4.8%) | 57 (40.7%) | <0.001 |
| **Lopinavir/Ritonavir** | 100% | 205 (21.9%) | 153 (19.2%) | 52 (37.4%) | 0.076 |
| **Azithromycin** | 100% | 606 (64.7%) | 539 (67.5%) | 67 (48.2%) | <0.001 |
| **Remdesivir** | 80% | 24 (3.2%) | 19 (3.1%) | 5 (3.9%) | 0.923 |
| **Chloroquine** | 100% | 701 (74.8%) | 596 (74.7%) | 105 (75.5%) | 0.244 |
| **Glucocorticoids** | 100% | 313 (33.4%) | 241 (30.2%) | 72 (51.8%) | 0.006 |
| **Tocilizumab** | 100% | 122 (13.0%) | 94 (11.8%) | 28 (20.1%) | <0.001 |

CKD, chronic kidney disease. COPD, chronic obstructive pulmonary disease. ILD, interstitial lung disease. G.I., gastrointestinal. ACE, angiotensin converting enzyme. NSAID, non-steroidal anti-inflammatory drugs. HDL, high density lipoprotein. LDL, low density lipoprotein. eGFR, estimated glomerular filtration rate. PaO_2_, partial oxygen pressure. IL-6, interleukin-6. **Mean time to discharge evaluated in patients discharged alive.

**Suppl. Table S5: Characteristics of subjects admitted to the Padua Hospital with COVID-19 and without AKI at admission, and factors associated with AKI onset after hospital admission.**

|  |  | No AKI during hospitalization  N=290 | AKI onset after admission  N=72 | Age-sex adjusted P |
| --- | --- | --- | --- | --- |
|  | avail | Value | Value |  |
| Age, years | 100% | 61.9 ± 15.1 | 72.2 ± 12.4 | **<0.0001** |
| Sex male, % | 100% | 155 (53.4%) | 53 (73.6%) | **0.001** |
| Concomitant Risk Factors, n (%) |  |  |  |  |
| Diabetes (Known+New) | 100% | 66 (22.8%) | 23 (31.9%) | 0.817 |
| Known Diabetes | 100% | 55 (19.0%) | 16 (22.2%) | 0.507 |
| Newly diagnosed Diabetes | 100% | 11 (3.8%) | 7 (9.7%) | 0.115 |
| Hypertension | 100% | 123 (42.4%) | 50 (69.4%) | 0.095 |
| Current smoking | 47% | 31 (23.8%) | 17 (42.5%) | 0.320 |
| Comorbidities, n (%) |  |  |  |  |
| Cardiovascular disease | 96% | 42 (14.8%) | 14 (21.5%) | 0.593 |
| Atrial fibrillation | 97% | 27 (9.5%) | 12 (17.9%) | 0.977 |
| CKD | 100% | 24 (8.3%) | 28 (38.9%) | **<0.0001** |
| COPD | 97% | 11 (3.9%) | 12 (17.9%) | 0.051 |
| Cancer | 96% | 39 (13.8%) | 18 (27.7%) | 0.196 |
| Symptoms at admission |  |  |  |  |
| Symptoms to hospitaliz, days | 90% | 7.3 ± 4.5 | 5.9 ± 4.1 | **0.044** |
| Body temperature (°C) | 46% | 38.5 ± 0.6 | 38.6 ± 0.6 | 0.354 |
| Cough, % | 89% | 175 (66.5%) | 34 (56.7%) | 0.281 |
| Dyspnea, % | 91% | 153 (57.5%) | 45 (71.4%) | 0.145 |
| Pneumonia / ILD, % | 93% | 231 (84.3%) | 57 (93.4%) | 0.728 |
| GI symptoms, % | 88% | 70 (27.1%) | 19 (31.7%) | 0.243 |
| Medication before hospitalization |  |  |  |  |
| ACE inhibitors, % | 100% | 39 (13.4%) | 22 (30.6%) | 0.077 |
| ARBs, % | 100% | 41 (14.1%) | 19 (26.4%) | 0.075 |
| Calcium channel blockers, % | 100% | 35 (12.1%) | 16 (22.2%) | 0.118 |
| Beta blockers, % | 100% | 51 (17.6%) | 17 (23.6%) | 0.638 |
| Anti-platelet agents, % | 100% | 41 (14.1%) | 16 (22.2%) | 0.887 |
| Statins, % | 100% | 52 (17.9%) | 17 (23.6%) | 0.600 |
| Oral Anticoagulants, % | 100% | 23 (7.9%) | 14 (19.4%) | 0.310 |
| Antibiotics, % | 100% | 97 (33.4%) | 26 (36.1%) | 0.859 |
| NSAID, % | 100% | 18 (6.2%) | 3 (4.2%) | 0.990 |
| Outcomes, n (%) |  |  |  |  |
| Oxygen | 100% | 207 (71.4%) | 69 (95.8%) | 0.010 |
| Non-invasive ventilation | 100% | 27 (9.3%) | 33 (45.8%) | <0.0001 |
| Invasive ventilation | 100% | 10 (3.4%) | 31 (43.1%) | <0.0001 |
| ICU | 100% | 21 (7.2%) | 34 (47.2%) | <0.0001 |
| ICU or death | 100% | 31 (10.7%) | 45 (62.5%) | <0.0001 |
| Death | 100% | 11 (3.8%) | 21 (29.2%) | <0.0001 |
| Discharged alive | 100% | 259 (89.3%) | 41 (56.9%) | <0.0001 |
| Mean days of hospitalization§ | 76% | 10.6 ± 6.5 | 18.2 ± 8.7 | <0.0001 |

Note: § Among survivors and truncated at 30-days;

**Suppl. Tables S6. Risk factors at admission and Hospital-acquired risk factors for AKI onset after hospital admission.** Data presented as mean (S.D.). (Analyses restricted to patients admitted to Padua-Italy center). Changes from baseline to worst value before AKI onset, only variable available in at least 40% of subjects are shown, Relative Risk (R.R.) per AKI onset per each increase in S.D. are shown only for variable with nominally significant association.

|  | **Risk factors at admission** | | | |  | **Hospital-acquired risk factors** | | | |
| --- | --- | --- | --- | --- | --- | --- | --- | --- | --- |
|  | Avail. | No AKI onset  N=290 | AKI onset after admission N=72 | Age-sex adjusted P |  | Avail. | **Changes prior to AKI** (N max =362) | R.R. of AKI onset per S.D. changes | Age-sex, and baseline adjusted P |
| Parameters at admission |  |  |  |  |  |  |  |  |  |
| Systolic BP, mm Hg | 99% | 129.2 ± 18.5 | 136.6 ± 21.0 | 0.422 |  | n.a. |  |  |  |
| Diastolic BP, mm Hg | 99% | 76.9 ± 11.4 | 78.1 ± 12.0 | 0.408 |  | n.a. |  |  |  |
| Heart rate, bpm | 98% | 85.8 ± 14.6 | 85.1 ± 16.3 | 0.414 |  | n.a. |  |  |  |
| Oxygen saturation, % | 99% | 95.3 ± 3.7 | 92.4 ± 5.8 | **0.002** |  | n.a. |  |  |  |
| P/F ratio | 68% | 301.6 ± 88.7 | 243.0 ± 100.7 | **0.025** |  | n.a. |  |  |  |
| *Predictors: Laboratory Results* |  |  |  |  |  |  |  |  |  |
| Serum creatinine, umol/l | 100% | 78.9 ± 19.2 | 115.3 ± 106.2 | **<0.0001** |  | 98% | 5.4 ± 10.7 | **1.12 (1.01-1.24)** | **0.033** |
| Potassium mmol/L | 100% | 3.9 ± 0.6 | 4.0 ± 0.6 | 0.522 |  | 96% | 0.4 ± 0.7 |  | 0.743 |
| Hematocrit % | 98% | 0.4 ± 0.1 | 0.4 ± 0.1 | 0.643 |  | 97% | 0.0 ± 0.1 |  | 0.484 |
| Hemoglobin, g/l | 99% | 136.2 ± 22.0 | 137.1 ± 20.3 | 0.736 |  | 98% | -15.5 ± 17.8 |  | 0.939 |
| Platelets, el/ul | 99% | 205.2 ± 85.8 | 222.4 ± 83.7 | **0.048** |  | 98% | -12.6 ± 54.7 |  | 0.447 |
| White blood cells, kel/µl | 99% | 10.7 ± 51.2 | 7.8 ± 4.2 | 0.359 |  | 98% | -0.3 ± 46.0 |  | 0.056 |
| Lymphocytes, kel/µl | 98% | 1.2 ± 1.1 | 1.1 ± 1.4 | 0.744 |  | 91% | 0.4 ± 0.8 |  | 0.565 |
| Neutrophils, kel/µl | 98% | 4.5 ± 2.9 | 6.0 ± 3.4 | **0.007** |  | 91% | 2.7 ± 4.9 |  | 0.254 |
| Eosinophils, kel/µl | 98% | 0.0 ± 0.1 | 0.0 ± 0.2 | 0.666 |  | 91% | 0.1 ± 0.1 |  | 0.123 |
| Monocytes, kel/µl | 98% | 0.5 ± 0.3 | 0.5 ± 0.4 | 0.657 |  | 91% | 0.4 ± 0.4 |  | 0.053 |
| C-reactive protein, mg/l | 99% | 63.7 ± 66.2 | 112.9 ± 82.5 | **<0.0001** |  | 96% | 24.0 ± 55.0 | **1.28 (1.07-1.54)** | **0.008** |
| IL-6, ng/l | 15% | 20 (9-49) | 49 (15-893) | **<0.0001** |  | n.a. |  |  |  |
| Ferritin, ug/L | 79% | 538 (238-993) | 808 (506-1347) | 0.106 |  | 46% | 250.6 ± 656 | **1.48 (1.14-1.93)** | **0.001** |
| Fibrinogen, ug/L | 74% | 4.6 ± 1.3 | 5.4 ± 1.7 | **<0.0001** |  | 56% | 0.4 ± 1.7 | **1.27 (1.05-1.54)** | **0.013** |
| Plasma glucose, mmol/l | 99% | 7.0 ± 3.2 | 7.7 ± 3.4 | 0.345 |  | 86% | 1.1 ± 3.2 | **1.33 (1.13-1.56)** | **0.001** |
| LDH, U/L | 97% | 325.9 ± 159.3 | 417.6 ± 209.4 | **0.006** |  | 82% | 10.5 ± 146.2 | **1.25 (1.03-1.51)** | **0.026** |
| Lactic Acid, mmol/l | 78% | 1.4 ± 0.6 | 1.5 ± 0.6 | 0.736 |  | 43% | 0.4 ± 1.1 | **1.24 (1.03-1.49)** | **0.025** |
| CPK, U/L | 91% | 153.4 ± 193.8 | 238.0 ± 279.8 | **0.033** |  | 62% | -16.5 ± 153 |  | 0.093 |
| Troponin I, ng/L | 59% | 107.7 ± 545.7 | 35.8 ± 56.8 | 0.134 |  | 41% | 331 ± 2346 |  | 0.345 |
| BNP, pg/ml | 37% | 97.0 ± 160.5 | 180.0 ± 202.9 | 0.500 |  | n.a. |  |  |  |
| AST, U/L | 98% | 43.2 ± 26.4 | 52.9 ± 30.7 | **0.027** |  | 92% | 20.8 ± 74.4 |  | 0.083 |
| ALT, U/L | 98% | 36.3 ± 28.6 | 37.7 ± 34.7 | 0.139 |  | 93% | 44.6 ± 120.8 | **0.36 (0.18-0.70)** | **0.003** |
| GGT, U/L | 98% | 54.9 ± 73.9 | 68.5 ± 103.1 | 0.064 |  | 79% | 33.0 ± 80.4 |  | 0.647 |
| Albumin g/L | 93% | 32.5 ± 5.1 | 30.1 ± 5.1 | 0.301 |  | n.a. |  |  | 0.192 |
| Bilirubin g/l | 96% | 10.1 ± 6.4 | 14.0 ± 33.2 | **0.014** |  | 77% | 4.5 ± 9.5 | **1.55 (1.38-1.74)** | **<0.0001** |
| Amylases U/L | 91% | 36.8 ± 19.8 | 45.0 ± 43.7 | **0.002** |  | 57% | 20.2 ± 50.8 | **1.10 (1.00-1.21)** | **0.044** |
| APTT | 81% | 1.0 ± 0.1 | 1.0 ± 0.2 | 0.962 |  | 67% | 0.01 ± 0.18 | **1.19 (1.11-1.27)** | **<0.0001** |
| INR | 86% | 1.2 ± 0.3 | 1.3 ± 0.6 | 0.447 |  | 73% | 0.0 ± 0.4 | **1.22 (1.04-1.43)** | **0.013** |
| D-dimer, ug/l | 95% | 179 (150-341) | 284 (156-576) | 0.122 |  | 77% | 44 (-4-351) |  | 0.898 |
| Art blood O2 saturation % | 57% | 93.7 ± 6.7 | 89.0 ± 13.9 | **0.002** |  | n.a. |  |  |  |
| Art blood PaO2 mmhg | 57% | 73.4 ± 34.0 | 67.3 ± 33.4 | 0.573 |  | n.a. |  |  |  |
| Art blood pH | 57% | 7.5 ± 0.0 | 7.5 ± 0.1 | 0.098 |  | n.a. |  |  |  |
| Art blood HCO3- mmol/l | 57% | 23.3 ± 3.0 | 22.9 ± 4.0 | 0.738 |  | n.a. |  |  |  |
| Art blood PaCO2 mmhg | 57% | 32.8 ± 4.6 | 33.3 ± 6.7 | 0.407 |  | n.a. |  |  |  |
| *Urine biochemical results* |  |  |  |  |  |  |  |  |  |
| Urine density | 89% | 1020.6 ± 8.2 | 1019.8 ± 7.1 | 0.297 |  | 41% | 0.4 ± 9.0 |  | 0.135 |
| Urine PH | 88% | 6.0 ± 0.7 | 5.8 ± 0.6 | 0.265 |  | 41% | 0.38 ± 0.84 | **0.47 (0.23-0.93)** | **0.031** |
| Urine Hb g/l | 80% | 0.001 ± 0.003 | 0.004 ± 0.004 | **0.001** |  | 40% | 0.00 ± 0.00 |  | 0.245 |
| Urine Protein g/l | 88% | 0.4 ± 0.5 | 0.7 ± 0.7 | 0.074 |  | 41% | -0.1 ± 0.6 |  | 0.107 |
| Urine Glucose mmol/l | 88% | 2.0 ± 9.2 | 3.1 ± 12.3 | 0.349 |  | 41% | 1.5 ± 11.9 |  | 0.558 |
| Urine Ketons g/l | 88% | 0.1 ± 0.2 | 0.0 ± 0.1 | 0.214 |  | 41% | 0.0 ± 0.2 |  | 0.299 |

Note: Avail: data available in % of patients. IL-6, interleukin-6; Art blood: arterial blood; n.a. data not avialble or available in less than 40% of subjects.

**Supp. Table S7: Effects of AKI on laboratory parameters during hospitalization and possible contributors to AKI-induced increased mortality risk in COVID-19.** Note: we analyzed changes from baseline or from AKI onset for those experiencing AKI, to worst in-hospital value. * P value are adjusted by Age-sex, and baseline-value. ° Possible contributors of AKI-induced increased mortality risk in COVID-19 are those both influenced by AKI and associated with mortality risk. Baseline model is adjusted by age, sex and CKD. The analyses were conducted in the imputed data set in order to allow a comparison across variable and in the same population. $: non estimable since more than 40% of subjects had missing value.

|  | Avail. | Mean overall changes from baseline or from AKI onset. |  | Effect of AKI on changes (in S.D.) | P * |  | Effect on risk of death  (R.R. and 95% C.I. per each S.D. increase) | P * |  | Contributors° |  | Association between AKI and death (RR 95% CI) After adjustment for changes in parameters | % of mediation |
| --- | --- | --- | --- | --- | --- | --- | --- | --- | --- | --- | --- | --- | --- |
|  |  |  |  |  |  |  |  |  |  |  |  | RR prior to adjustment for changes: 3.04 (1.54-6.01) |  |
| Parameters |  |  |  |  |  |  |  |  |  |  |  |  |  |
| Systolic BP, mm Hg | 99% | -19.8 ± 19.5 |  |  | 0.544 |  |  | 0.350 |  |  |  |  |  |
| Diastolic BP, mm Hg | 99% | -11.0 ± 12.9 |  |  | 0.146 |  |  | 0.483 |  |  |  |  |  |
| Heart rate, bpm | 98% | 7.8 ± 16.6 |  |  | 0.053 |  | **↗ 1.30 (1.01-1.69)** | **0.043** |  |  |  |  |  |
| Oxygen saturation, % | 99% | -2.7 ± 5.3 |  | **↘ -0.4 S.D. (0.1)** | **0.001** |  | **↘ 0.79 (0.66-0.94)** | **0.007** |  | **X** |  | **2.27 (1.14-4.52)** | **26%** |
| P/F ratio | 68% | -61.0 ± 95.6 |  | **↘ -0.5 S.D. (0.2)** | **0.005** |  | **↘ 0.43 (0.26-0.70)** | **0.001** |  | **X** |  | **1.89 (0.81-4.44)** | **43%** |
| *Laboratory Results* |  |  |  |  |  |  |  |  |  |  |  |  |  |
| Serum creatinine, umol/l | 99% | 15.1 ± 43.9 |  | **↗ 1.1 S.D. (0.1)** | **<0.0001** |  | **↗ 1.34 (1.22-1.48)** | **<0.0001** |  | **X** |  | **2.36 (1.18-4.72)** | **23%** |
| Potassium mmol/L | 96% | 0.5 ± 0.7 |  | **↗ 0.5 S.D. (0.1)** | **<0.0001** |  |  | 0.365 |  |  |  |  |  |
| Hematocrit % | 96% | -0.05 ± 0.07 |  |  | 0.136 |  |  | 0.195 |  |  |  |  |  |
| Hemoglobin, g/l | 96% | -15.9 ± 17.9 |  |  | 0.259 |  |  | 0.091 |  |  |  |  |  |
| Platelets, el/ul | 96% | -17.2 ± 65.7 |  | **↘ -0.5 S.D. (0.1)** | **<0.0001** |  | **↘ 0.67 (0.48-0.94)** | **0.021** |  | **X** |  | **2.94 (1.46-5.89)** | **3%** |
| White blood cells, kel/µl | 96% | -0.1 ± 46.3 |  | **↗ 0.1 S.D. (0.0)** | **0.002** |  |  | 0.099 |  |  |  |  |  |
| Lymphocytes, kel/µl | 93% | 0.6 ± 1.1 |  | **↗ 0.6 S.D. (0.1)** | **<0.0001** |  |  | 0.274 |  |  |  |  |  |
| Neutrophils, kel/µl | 93% | 2.6 ± 5.1 |  |  | 0.349 |  |  | 0.457 |  |  |  |  |  |
| Eosinophils, kel/µl | 93% | 0.1 ± 0.2 |  | **↗ 0.5 S.D. (0.1)** | **0.001** |  |  | 0.315 |  |  |  |  |  |
| Monocytes, kel/µl | 93% | 0.4 ± 0.5 |  |  | 0.894 |  | **↘ 0.61 (0.41-0.90)** | **0.014** |  |  |  |  |  |
| C-reactive protein, mg/l | 96% | 24.3 ± 58.5 |  |  | 0.123 |  | **↗ 1.41 (1.07-1.85)** | **0.013** |  |  |  |  |  |
| Ferritin, ug/L | 46% | 217.5 ± 669.2 |  |  | 0.357 |  | **↗ 2.39 (1.60-3.34)** | **<0.0001** |  |  |  |  |  |
| Fibrinogen, ug/L | 57% | 0.5 ± 1.8 |  | **↗ 0.5 S.D. (0.2)** | **0.009** |  |  | 0.087 |  |  |  |  |  |
| Plasma glucose, mmol/l | 80% | 0.9 ± 3.2 |  | **↗ 0.5 S.D. (0.2)** | **0.005** |  |  | 0.320 |  |  |  |  |  |
| LDH, U/L | 82% | 37.3 ± 406.8 |  | **↗ 0.5 S.D. (0.2)** | **0.002** |  | **↗ 1.23 (1.18-1.28)** | **<0.0001** |  | **X** |  | **2.67 (1.44-4.96)** | **12%** |
| Lactate Acid, mmol/l | 42% | 0.5 ± 1.4 |  | **↗ 0.6 S.D. (0.2)** | **0.006** |  | **↗ 1.30 (1.01-1.68)** | **0.045** |  | **X** |  | **3.02 (1.61-5.68)** | **1%** |
| CPK, U/L | 63% | -13.5 ± 164.7 |  | **↗ 0.4 S.D. (0.2)** | **0.020** |  |  | 0.327 |  |  |  |  |  |
| Troponin I, ng/L | 43% | 450.3 ± 2665.0 |  |  | 0.276 |  | **↗ 1.31 (1.31-1.70)** | **<0.0001** |  |  |  |  |  |
| AST, U/L | 91% | 30.3 ± 129.0 |  |  | 0.213 |  | **↗ 1.12 (1.05-1.20)** | **<0.0001** |  |  |  |  |  |
| ALT, U/L | 92% | 54.1 ± 131.0 |  |  | 0.890 |  |  | 0.478 |  |  |  |  |  |
| GGT, U/L | 81% | 44.4 ± 118.0 |  | **↗ 0.6 S.D. (0.2)** | **<0.0001** |  |  | 0.263 |  |  |  |  |  |
| Bilirubin g/l | 79% | 5.3 ± 33.8 |  |  | 0.058 |  |  | 0.731 |  |  |  |  |  |
| Amylases U/L | 60% | 18.0 ± 50.0 |  |  | 0.781 |  | **↗ 1.48 (1.07-2.06)** | **0.017** |  |  |  |  |  |
| Anti-thrombin III % | 16% | 10.5 ± 16.8 |  |  | 0.487 |  |  | 0.867 |  |  |  |  |  |
| APTT | 66% | 0.03 ± 0.31 |  | **↗ 0.5 S.D. (0.2)** | **0.003** |  | **↗ 1.21 (1.14-1.28)** | **<0.0001** |  | **X** |  | **2.92 (1.54-5.57)** | **3%** |
| INR | 72% | 0.0 ± 0.3 |  | **↗ 0.7 S.D. (0.2)** | **<0.0001** |  | **↗ 1.29 (1.03-1.62)** | **0.027** |  | **X** |  | **2.97 (1.52-5.81)** | **2%** |
| D-dimer, ug/l | 78% | 60 (-4-419) |  |  | 0.986 |  |  | 0.230 |  |  |  |  |  |
| *Urine biochemical results* |  |  |  |  |  |  |  |  |  |  |  |  |  |
| Urine density | 43% | 1.0 ± 7.8 |  | **↗ 0.2 S.D. (0.2)** | **0.001** |  | **↗ 2.00 (1.07-3.72)** | **0.029** |  | **X** |  | n.e.$ |  |
| Urine PH | 43% | 0.49 ± 0.92 |  |  |  |  |  | 0.585 |  |  |  |  |  |
| Urine Hb g/l | 42% | 0.001 ± 0.003 |  | **↗ 0.6 S.D. (0.2)** | **<0.0001** |  |  | 0.949 |  |  |  |  |  |
| Urine Protein g/l | 43% | -0.05 ± 0.56 |  | **↗ 0.1 S.D. (0.2)** | **<0.0001** |  | **↗ 1.78 (1.04-3.04)** | **0.037** |  | **X** |  | n.e.$ |  |

**Supplementary Figures:**

**Figure S1: Association between AKI and death according to patients’ characteristics.** Note: ** indicates nominally significant interaction (p<0.05), i.e. the association between AKI and death is different in the two groups. N.B. Cutoff for SpO2, Hematocrit, White Blood Cell and platelet count, C-reactive Protein and D-Dimer were evaluated accordingly to top quartile of the distribution of each variable. (Analyses adjusted by age, sex and clinical center).

**
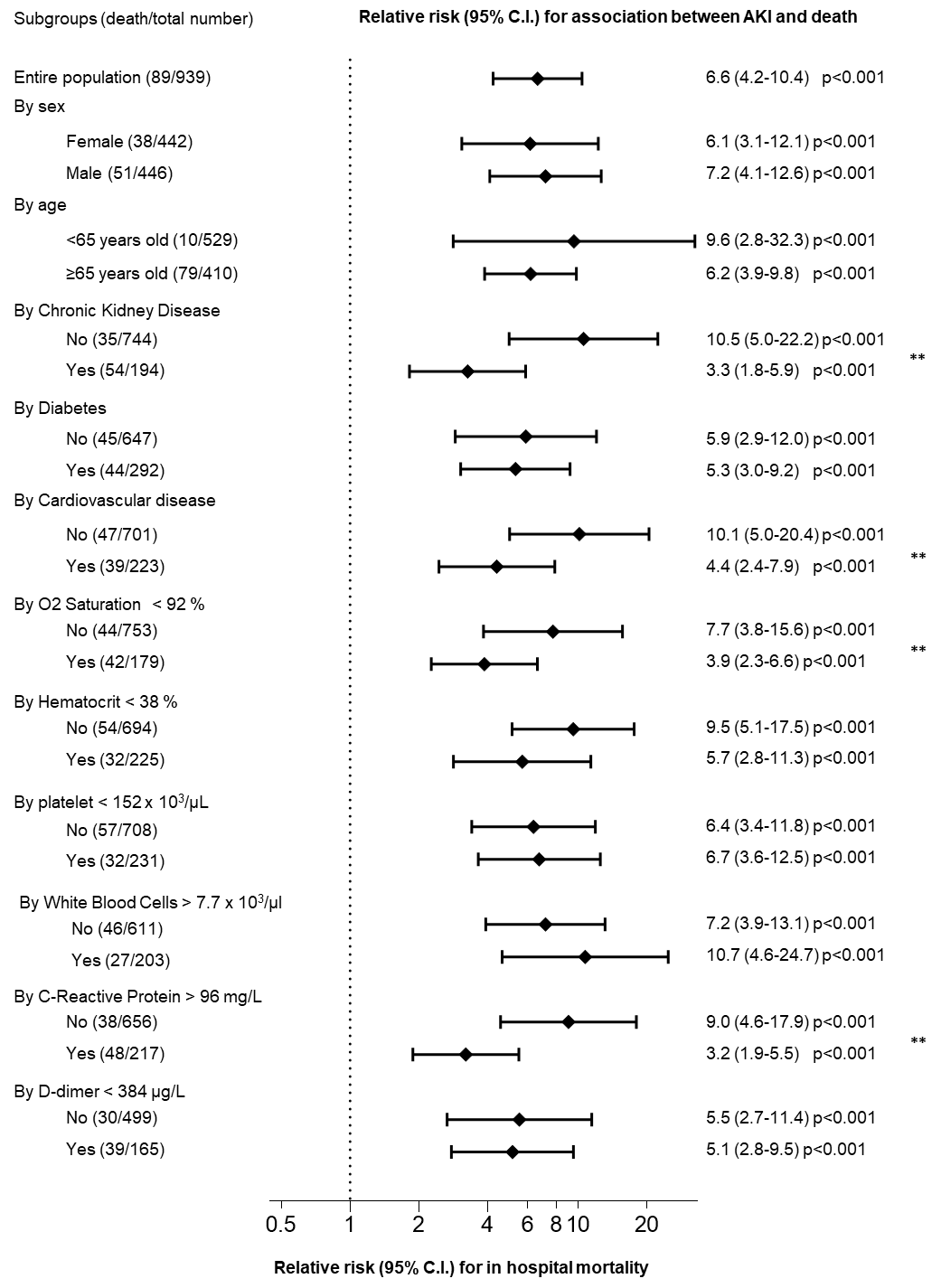
**
